# Changing the Lives of People With Primary Immunodeficiencies (PI) With Early Testing and Diagnosis

**DOI:** 10.3389/fimmu.2018.01439

**Published:** 2018-06-25

**Authors:** Antonio Condino-Neto, Francisco J. Espinosa-Rosales

**Affiliations:** ^1^Department of Immunology, Institute of Biomedical Sciences, Institute of Tropical Medicine, University of São Paulo, São Paulo, Brazil; ^2^Latin American Society for Primary Immunodeficiencies, Santiago, Chile; ^3^Fundación Mexicana para Niñas y Niños con Inmunodeficiencias (FUMENI), Mexico, Mexico

**Keywords:** primary immunodeficiencies, diagnosis, treatment, morbidity, mortality

Throughout the world, primary immunodeficiencies (PI) are largely undiagnosed and underreported. Many patients do not receive an accurate and timely diagnosis, which is crucial for successful management and care of PI (1). It is estimated that between 70 and 90% of people living with a PI still remain undiagnosed worldwide (2). As for many other rare diseases, early testing and diagnosis are essential first steps in the care pathway toward timely and appropriate care and treatment, which are ultimately life changing and life enhancing for people living with PI.

Under the theme “My future starts with early testing and diagnosis of PI,” the eighth annual World Primary Immunodeficiency Week (World PI Week), celebrated from the 22nd to the 29th of April 2018, raised awareness of early recognition of PI, pursuing the goal of the campaign of ensuring an improved quality of life for people living with these diseases worldwide.

## Introduction to PI

Classified as “rare diseases,” PI are a group of inherited and genetic defects of the immune system, which is partly or totally missing or does not function properly. These deficiencies make people with PI more prone to a wide range of infections affecting different parts of the body such as skin infections, sinopulmonary infections, infections of the intestines, etc; and are often chronic and debilitating (3). PI also results in an increased susceptibility to autoimmune disorders, autoinflammatory diseases, allergy diseases, and cancer, in parallel with recurring infections. While antibody deficiencies are the most commonly diagnosed types of PI, over 350 forms of PI exist (4). They can appear at any age, but the more severe forms of the disease are more frequent in infancy or early childhood. Because PI often present themselves in the form of infections, with a variety of symptoms and clinical manifestations, practitioners often treat the infections while missing the underlying cause. This frequently leads to reoccurrence of infections and persistence of the condition, leaving the individual vulnerable to health deterioration, physical disability, permanent organ damage, or even death. Estimates indicate that from symptom onset to diagnosis of a PI it takes an average of 12.4 years in the US (5). This means that many people with PI experience recurring infections with negative consequences that can affect their personal, social, and professional life for over a decade. However, once recognized and treated, patients can lead normal, productive lives (3).

## PI Diagnosis

The first step in diagnosing PI consists of a detailed evaluation of the immune system, which includes reviewing medical and family history, physical examination, and blood and/or vaccine tests. Several types of testing exist, and the initial testing is usually performed *via* blood testing, followed by further tests of the immune system such as blood cell tests (e.g., complete blood count with a differential count of the white blood cells); and measurement of immunoglobulin levels. When a family history of PI or PI-like symptoms exist, PI can be detected more easily in newborns or children, while in other cases patients live with the symptoms and recurring infections for years before the correct tests are done to diagnose the disease.

Due to the complex nature of PI and the fact that this group of diseases is caused by defects in genes that are involved in the development and functioning of the immune system (6), genetic testing is often required to ensure the best diagnosis possible. Latest advances in genetic technology have contributed significantly to the diagnosis of PI (3); however, genetic testing is not available in many countries, in particular in the developing world. This is one of the challenges that limit early diagnosis of the disease and thus provision of appropriate and timely care. In the case of severe combined immunodeficiency (SCID), newborn screening is a cost-effective method to drive early diagnosis of the disease. The onset of clinical manifestations tends to occur usually before the third month of life and therefore better opportunities for cure depends on an early stem cell transplantation, before the patient becomes infected (7). Recent studies also indicate that newborn screening dramatically improves the outcomes of children with SCID (3). Though a comprehensive report on the status of newborn screening worldwide published in 2015 reflects the great variability in screening practices in different regions (8).

Although reasons vary according to regions and countries of the world, delays in early PI diagnosis can be explained by a perception that recurring infections are part of a child’s normal development; by the difficulty for non-immunologists to identify PI within the wide range of potential cases and to decide when to start further investigating a patient for PI; by an increasing number of types of PI being recognized; by a lack of diagnostic and testing facilities and patient access to them as well as to specialist care, resulting in unnecessary complications; by a relatively low level of awareness of symptoms of PI among primary care doctors (general practitioners and pediatricians) and within the population (9); and by the low awareness of professional guidelines for diagnosis and management of PI among family practice physicians. This said, the awareness of PI in certain regions of the world is slowly increasing.

A questionnaire was recently sent to countries that participate in the Latin American Society for Immunodeficiencies registry, with questions related to PI challenges in each participating country. The results led to the conclusion that the greatest challenge is the availability of laboratory tests to investigate newly described PI (10).

It is important to note that despite those challenges, a continued increase in the number of patients with PI identified, diagnosed, and treated is reported in a recent study based on physician-reported data (9).

## PI in Health Policy-Making

Diagnostic delays are not only damaging to the patient with a deterioration of his/her condition and difficult for the family caring for their relative, they also have a negative impact on healthcare systems due to inappropriate use of health resources caused by avoidable visits to a variety of different specialists for recurring infections (3). Conversely, prompt diagnosis of PI leads to better use of healthcare facilities (11) and has been demonstrated to generate lower healthcare costs (12). A 2009 peer-reviewed study documenting the financial impact associated with early diagnosis and management of PI in the US (13) found that costs to the healthcare system for each undiagnosed patient with an underlying PI amount to an average of $102,552 (€75,587.40) annually, while diagnosis and treatment yielded average savings of $79,942 (€589,223) per patient per year.

The exact prevalence of PI in the general population is still unknown (7), and it remains unclear whether the prevalence and incidence of the diseases have been evaluated accurately. PI may be more common than previously estimated, as outlined by recent studies (14). Estimates of the frequency of PI need to be revised regularly in accordance with the discovery of new types of PI. Therefore, it is important to conduct proper studies at country level to assess the proportion of affected individuals among the general population more efficiently and systematically. National registries are also valuable tools to achieve this aim (3), as well as to detect areas of low-diagnostic rates and to provide insight on diagnostic delays associated with increased morbidity and mortality. The important information they provide should guide health policy-making and be used by Governments to tailor national policies to the needs. Registries should be established in all countries and regions of the world to provide robust data and be sustained by international or cross-border registries due to the scarcity of some rare forms of PI.

## Awareness of PI

Greater public awareness of symptoms, as well as increased education among both primary and specialist care providers at pre- and postgraduate levels, are paramount for PI recognition so that healthcare professionals learn to recognize the different patterns of clinical presentation of the diseases. A recent study conducted in Finland showed that increased awareness of CVID among physicians would likely lead to earlier diagnosis and improved quality of care (15). The role of awareness campaigns, patient organizations, and scientific societies are fundamental in providing educational guidance and driving understanding (3). The relevance of education and warning campaigns to increase PI recognition, diagnosis, and treatment is also particularly stressed in the recent global report on primary immunodeficiencies, 2018 update (9). It has also been observed that there is a need to develop protocols for diagnosing PI that do not require extensive knowledge of the immune system to allow use by non-immunologists and make the recognition of the diseases easier. Improved training of immunologists, as their advice is extremely important during the diagnostic process, and appropriate training of medical students in immunology are required to facilitate diagnosis. Political and financial factors play a decisive role in educational efforts aimed at the medical and nursing communities (3).

## Conclusion

In order to ensure testing and diagnosis early on and diagnose unknown forms of the disease, a number of priorities can be identified:

– developing better diagnostic facilities and guaranteeing patients access to them (3);
– ensuring access to screening tests for severe antibody deficiencies to the whole range of hospital doctors and primary care providers;
– implementing widely routine newborn screening programs for severe forms of PI, including SCID (16), in both public and private healthcare settings;
– upscaling screening in patients with recurrent infections, irrespective of age and in a patient-centered approach;
– ensuring patient access to genetic testing and widespread availability in all medical specialties.

Several initiatives have been undertaken within the scientific, patient, and advocacy communities to address the challenges linked with PI recognition and ultimately reduce diagnosis delays. These include raising awareness of the warning signs of PI—the strongest warning sign being positive family history—in children (0–18 years old) and adults, both among primary care physicians and the general public. “Red Flags” (17) lists have been developed to alert and educate healthcare professionals about the possibility of PI and coordinate further testing and investigation or urgent referral to an immunologist where needed. Efforts have also been made to develop warning signs for PI among different medical specialties (18), and a multi-stage diagnostic protocol for screening of PI designed for non-immunologists (19) intend to increase PI awareness among adult physicians and pediatricians working in different fields, for use throughout Europe. Other guidelines to enhance earlier diagnosis include the Diagnostic & Clinical Care Guidelines for PID (20), which provide practical information for patients and healthcare providers about the functions of the immune system and PI, genetic counseling, and health insurance.

Furthermore, a 2004 study developed a method using an ICD-9-based scoring algorithm to identify PID patients in an urban hospital database, concluding in the effectiveness of the coding to “identify undiagnosed minority patients with immunodeficiency” (21). The SPIRIT^®^ (Software for Primary Immunodeficiency Recognition, Intervention, and Tracking) Analyzer also aims to alert physicians of medium and high-risk patients with recurring infections, by matching 352 ICD-10 codes to the 10 warning signs of PI to identify at-risk patients (9).

Timely diagnosis and appropriate treatment remain the keys to the successful management of people with PI (7). They prevent complications of infections and can allow one to live as normal of a life as the general population. While diagnosis and treatment can be complex, patient-centricity in the care setting, referral to further investigation upon any suspicion of PI, greater awareness of regionally targeted warning signs of PI toward the general public, physicians, pediatricians, and other medical specialists, and systematic education on the immune system for non-specialized healthcare professionals are paramount to saving and enhancing the lives of people with PI.

Healthcare authorities, governments, and state agencies in all countries should work in collaboration with public and private healthcare providers, scientific organizations, experts, and patient organizations to define and setup the healthcare policies and financial measures needed to facilitate an early diagnosis and ensure patients’ access to the services that will help them get their PI identified and treated, as well as manage the impact of the diseases on their lives.

## Author Contributions

Both authors planned, wrote, and revised the manuscript.

## Conflict of Interest Statement

The authors declare that the research was conducted in the absence of any commercial or financial relationships that could be construed as a potential conflict of interest.
